# The Antiviral Drug Valacyclovir Successfully Suppresses Salivary Gland Hypertrophy Virus (SGHV) in Laboratory Colonies of *Glossina pallidipes*

**DOI:** 10.1371/journal.pone.0038417

**Published:** 2012-06-05

**Authors:** Adly M.M. Abd-Alla, Henry Adun, Andrew G. Parker, Marc J.B. Vreysen, Max Bergoin

**Affiliations:** 1 Insect Pest Control Laboratory, Joint FAO/IAEA Division of Nuclear Techniques in Food and Agriculture, Vienna, Austria; 2 Laboratoire de Pathologie Comparée, Université Montpellier, Montpellier, France; University of Illinois at Chicago, United States of America

## Abstract

Many species of tsetse flies are infected with a virus that causes salivary gland hypertrophy (SGH) symptoms associated with a reduced fecundity and fertility. A high prevalence of SGH has been correlated with the collapse of two laboratory colonies of *Glossina pallidipes* and colony maintenance problems in a mass rearing facility in Ethiopia. Mass-production of *G. pallidipes* is crucial for programs of tsetse control including the sterile insect technique (SIT), and therefore requires a management strategy for this virus. Based on the homology of DNA polymerase between salivary gland hypertrophy virus and herpes viruses at the amino acid level, two antiviral drugs, valacyclovir and acyclovir, classically used against herpes viruses were selected and tested for their toxicity on tsetse flies and their impact on virus replication. While long term *per os* administration of acyclovir resulted in a significant reduction of productivity of the colonies, no negative effect was observed in colonies fed with valacyclovir-treated blood. Furthermore, treatment of a tsetse colony with valacyclovir for 83 weeks resulted in a significant reduction of viral loads and consequently suppression of SGH symptoms. The combination of initial selection of SGHV-negative flies by non-destructive PCR, a clean feeding system, and valacyclovir treatment resulted in a colony that was free of SGH syndromes in 33 weeks. This is the first report of the use of a drug to control a viral infection in an insect and of the demonstration that valacyclovir can be used to suppress SGH in colonies of *G. pallidipes*.

## Introduction

In sub-Saharan Africa, tsetse flies (*Glossina* spp.) are the vectors of two debilitating diseases, human sleeping sickness and the cattle disease nagana [1] and are a major constraint to the development of sustainable livestock production systems and the reduction of hunger and poverty [2]. Due to the lack of vaccines, the difficulty of drug treatment for sleeping sickness, and the development of resistance to the available trypanocidal drugs for nagana [3], vector control remains the most efficient strategy for the sustainable management of these diseases [4].

The successful eradication of *Glossina austeni* from Unguja Island (Zanzibar) using an area-wide integrated pest management approach including the sterile insect technique (SIT) (1994–1997) [5] encouraged several African countries to incorporate the SIT in their national tsetse control programs. A facility to produce sterile flies for SIT application in the Southern Rift Valley of Ethiopia was inaugurated in 2007 [6]. A *Glossina pallidipes* colony was initiated with wild flies from the target area but colony build-up was hampered due to the high prevalence (26–40%) of salivary gland hypertrophy (SGH) syndrome in the flies [7], [8]. Similar cases were reported at the FAO/IAEA Insect Pest Control Laboratory, Seibersdorf, Austria where 2 colonies of *G. pallidipes* with a SGH prevalence up to 85% collapsed in 1987 and 2001. *G. pallidipes* flies with SGH have no or reduced progeny and could jeopardize eradication programs with an SIT component against this species.

The causative agent of SGH, the *G. pallidipes* salivary gland hypertrophy virus (GpSGHV), is a nuclear, rod-shaped, enveloped dsDNA virus averaging 70×640 nm in size that infects various tissues in tsetse but hypertrophy symptoms appear only in salivary glands [9]–[15]. The virus in wild tsetse populations is transmitted vertically from mother to progeny either by transovum transmission or to developing larvae through infected milk glands, whereas under laboratory conditions horizontal transmission of the virus through the *in vitro* blood feeding technique is the main source of infection [8], [16]. As much as 10^7^ virus particles per ml can be deposited by a single heavily infected fly via salivary secretions into the blood and this virus could easily be taken up by other, uninfected flies [8]. These data, along with the complete sequencing of the viral genome [17] provided guidance to potential strategies for controlling GpSGHV in tsetse laboratory or mass-reared colonies [8]. Several strategies are under investigation including (i) clean feeding where each cage of flies is offered unused (fresh) blood, and (ii) commercial antiviral drugs added to the blood diet to reduce virus replication [18].

The discovery in the late 1970s that acyclic nucleoside analogues, in particular acyclovir, could inhibit DNA replication of herpes simplex virus (HSV) at concentrations far below those that affect cellular DNA synthesis sparked a new era in antiviral chemotherapy [19]. The underlying reason for the selectivity – that acyclovir is specifically converted to the active metabolite by an HSV-encoded thymidine kinase – was unexpected but the potential for exploiting viral enzymes to develop potent and specific antiviral drugs was clearly demonstrated. Acyclovir subsequently became a successful treatment for HSV-1 and HSV-2 [19]–[21]. Later, many antiviral drugs that inhibit the DNA polymerase of herpes viruses were developed and tested. After completing the genome sequence of GpSGHV, phylogenetic analysis of its DNA polymerase amino acid sequence revealed that it shares the highest similarity with herpes virus DNA polymerase [17]. These similarities led us to speculate that the antiviral drugs against herpes viruses might inhibit or reduce GpSGHV replication and prompted research to assess the feasibility of using these antiviral drugs to manage the GpSGHV infection in colonies of *G. pallidipes*. Here we present the results of evaluating two antiviral drugs, acyclovir and valacyclovir, currently used against herpesviruses, for their ability to suppress viral infection and SGH symptoms in colonies of *G. pallidipes*. Valacyclovir is the L-valyl ester of acyclovir and has shown to have a better bioavailability and oral absorption in HSV treatments [19]–[21].

## Results and Discussion

### Effect of Valacyclovir and Acyclovir on the Fly Biology

**Figure 1:**
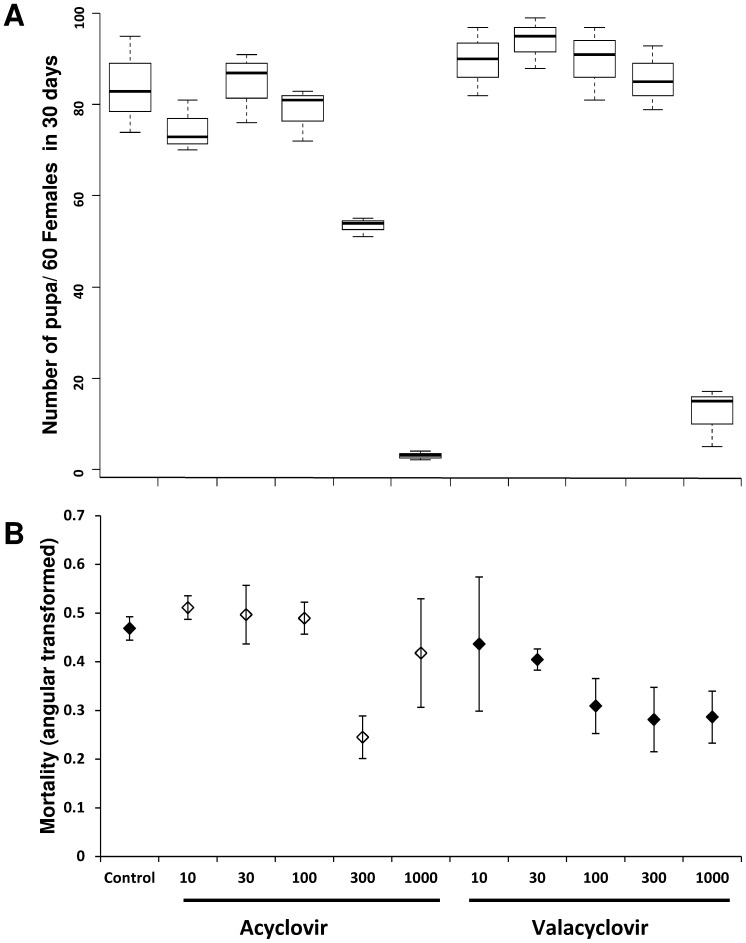
Dose effect of Acyclovir and Valacyclovir on tsetse flies *G. pallidipes* productivity and mortality. Flies were fed on blood supplemented with different concentrations (10 to 1000 µg/ml) of the two antiviral drugs for 60 days and the produced pupae (A) and mortality (B) were recorded. Flies fed with drug-free blood were used as control.

The impact of valacyclovir and acyclovir when given orally in the blood diet on tsetse fly reproduction and survival was tested at different concentrations (10–1000 µg/ml) of each of the two drugs in Experiment 1. After treatment, fly fecundity and mortality were checked. The highest concentration (1000 µg/ml) of both acyclovir and valacyclovir significantly reduced the fecundity expressed as the number of pupae produced per initial female (PPIF) **(** **Fig. 1A** **)**. At concentrations lower than 1000 µg/ml, no significant differences were observed in PPIF between the drug-treated and non-treated flies (*P* = 0.348), nor among the different concentrations of the drugs (*P* = 0.104). The mortality rate after 60 days of acyclovir-treated flies averaged 20% regardless of the drug concentration, with the unexplained exception of flies treated with 300 µg/ml where a significantly lower mortality was recorded **(** **Fig. 1B** **)**. Conversely, the mortality rate after 60 days in valacyclovir-treated flies decreased proportionally with increasing drug concentration. While at low concentration (10 µg/ml) mortality in the treated flies averaged 20% after 60 days, at the highest concentration (1 mg/ml) only 8% mortality was recorded (**Fig 1B**). Similar to the productivity, no significant differences in mortality were observed between antiviral drug treated and non-treated flies (*P* = 0.149) or among the different concentrations of the drugs (*P* = 0.118). Based on these data, the 300 µg/ml concentration appeared to be the most efficient concentration to block virus replication and was selected for subsequent experiments.

### Effect of Valacyclovir and Acyclovir Treatment on SGHV Load and SGH Prevalence

**Figure 2:**
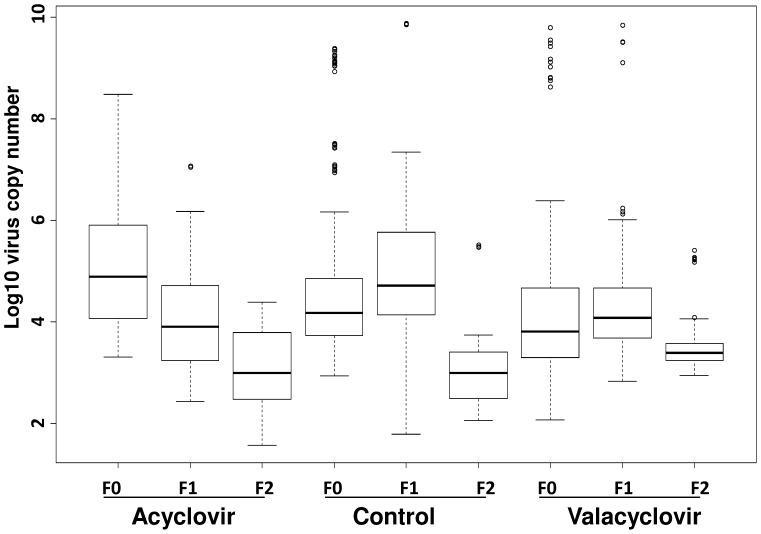
Effect of antiviral drug treatments in combination with clean feeding on the SGHV load in tsetse flies over three generations. At each generation flies were fed on blood supplemented with 300 µg/ml antiviral drug for 60 days, then virus load was estimated by qPCR as previously reported [22]. Flies fed with drug-free blood were used as control.

In experiment 2, the impact of the two drugs on SGHV replication was analyzed by feeding samples of 60 flies (45 females and 15 males) in three replicates with blood containing 300 µg/ml of acyclovir or valacyclovir for three successive fly generations in combination with clean feeding and measuring the virus loads by qPCR. A significant reduction (*P*<0.0001) in virus load was observed between generations following both treatments **(** **Fig. 2** **)**. Virus loads in all F2 generation flies treated either with acyclovir or valacyclovir were clearly under the threshold of 10^9^ copy number measured for flies with hypertrophied salivary gland [22]. A similar reduction in virus load was however detected in the control flies in the F2, consistent with our previous report of a reduced virus load in flies fed only on fresh blood using clean feeding for three generations [8].

**Figure 3:**
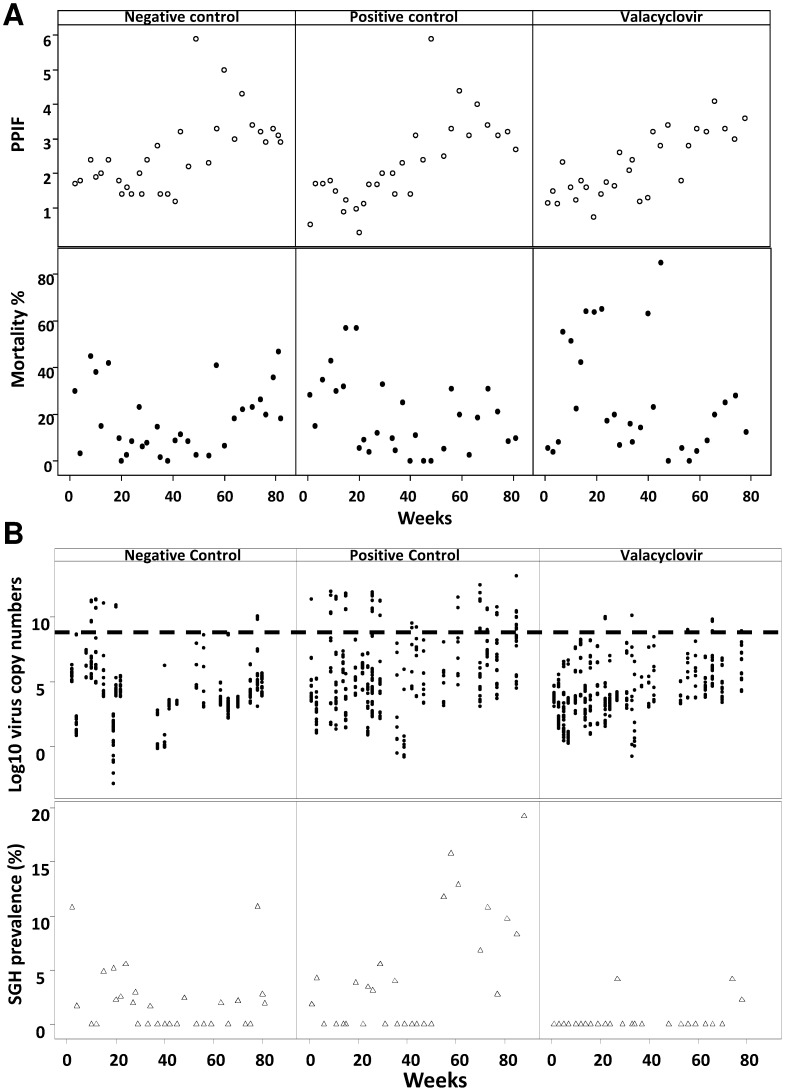
Effect of long term treatment with valacyclovir on *G. pallidipes* biology and virus prevalence. (**A**) Productivity (PPIF) and mortality of flies fed on blood supplemented with 300 µg/ml antiviral drug for 60 days**.** (**B**) SGHV virus load and SGH prevalence by qPCR and dissection, at 60 days after feeding. (**---**): threshold virus load correlated with SGH symptoms. Negative control: flies fed exclusively on clean blood; positive control: Flies fed on virus contaminated blood; valacyclovir: flies fed on virus contaminated blood with 300 µg/ml valacyclovir.

To further distinguish which of the two factors, antiviral drug or clean feeding, was responsible for the reduction of the virus load, 3 groups of *G. pallidipes* flies with initial low viral load (as measured according to [22]) were maintained for 19 months either under clean feeding conditions or on blood deliberately contaminated with virus and supplemented or not with the antiviral drugs (experiment 3). During the experiment we observed that long term feeding of the flies with blood supplemented with acyclovir progressively reduced fly fecundity (PPIF) over time. The group of acyclovir-treated flies was, therefore, removed from the experiment after 28 weeks. In the group fed with blood supplemented with valacyclovir, acceptable rates of productivity (*P* = 0.432) and mortality (*P* = 0.162) were maintained through several generations without a significant difference between treated and non-treated flies **(** **Fig. 3A** **)**.

With respect to the impact of long term treatment with valacyclovir on SGHV replication, we observed a significant reduction in SGH symptoms (*P*<0.001) (**Table 1** **)** to an overall average of 0.63% (4/633, from 24 samples) after 83 weeks of the treatment whereas in the untreated group fed on contaminated blood, SGH prevalence averaged 5.62% (43/765) with a maximum of 19% **(** **Fig. 3B** – positive control**)**. The application of clean feeding alone decreased SGH prevalence to an average of 1.9% (22/1154) (**Fig. 3B**) negative control). These results clearly demonstrated that valacyclovir alone suppressed GpSGHV replication (*P*<0.001) and maintained the virus infection far below the threshold level of 10^9^ viral copies required to develop SGH in *G. pallidipes* [22]. The overall effect of valacyclovir on GpSGHV replication mimics that of this drug in controlling herpesvirus infections in humans. It suppresses the symptom of SGH but does not eradicate the virus from the colony. This suggests a similar mode of action leading to the specific inhibition of the GpSGHV DNA polymerase through a pathway which remains to be elucidated. In contrast to herpesviruses, no gene homologous to a thymidine kinase has been identified in the GpSGHV genome [17]. The prodrug is, thus, very likely phosphorylated by a cellular thymidine kinase assumed to be particularly abundant in infected cells where active viral DNA replication occurs. The absence of negative side effects of the drug on productivity (*P* = 0.432) and survival (*P* = 0.162) of *G. pallidipes* during long term administration (2.5 years) supports the idea of using the drug for large-scale rearing of *G. pallidipes*. Moreover, the relatively cheap price of this drug compared to the high cost of clean feeding makes its application economically attractive. The reduced fecundity of flies subjected for an extended period to acyclovir treatment only is difficult to explain, especially since we previously reported that most of the valacyclovir ingested by the flies is converted to acyclovir [23], as is the case for vertebrates. In vertebrates, this conversion is known to occur rapidly in both the intestine and liver. In contrast nothing is known on the site and rate at which the cleavage of valacyclovir occurs in tsetse flies. The availability of acyclovir in the flies and the peak haemolymph concentration will depend on the concentration fed, the rate of uptake, breakdown and excretion whereas the availability of the active moiety (acyclovir) in the flies fed valacyclovir will also depend on the rate of conversion of valacyclovir to acyclovir. It might be speculated that the availability of a high concentration of acyclovir can have a negative impact on host DNA replication, especially in the embryonic phase when a high rate of DNA replication is required for cell division. Blockage of the DNA polymerase activity in this embryonic phase will affect the flies’ productivity. A possible explanation of the lack of these negative side effects in valacyclovir-treated flies is that the conversions rate of valacyclovir to acyclovir probably maintains the peak haemolymph concentration at a lower level than in the acyclovir treatment.

**Table 1:** Effect of valacyclovir on the incidence of salivary gland hypertrophy in *Glossina pallidipes,* analysis of variance and pairwise comparison of treatment means.

	Mean^a^	Variance	Detransformed mean
Valacyclovir	0.022499	0.003948	0.000506
Positive control	0.15367	0.023355	0.023429
Negative control	0.100026	0.011388	0.009972

	*V*	*P*	*N*
*V^b^*		**	*
*P*	5.87*^c^*		n.s.
*N*	3.52	2.51	

F = 8.66.

d.f. = 2, 80.

P = 0.00039.

T values for comparison of means.

### Effect of Combination of Valacyclovir and Clean Feeding on SGH Prevalence

Previous work had shown that the virus load in asymptomatic and symptomatic females flies determined to a large extend the prevalence of symptomatic infection in their progeny (2) and that flies with SGH excrete several orders of magnitude more virus particles when feeding than asymptomatic flies [8]. Furthermore, results of experiments 2 and 3 indicated that valavcyclovir treatment whether alone or combined with clean feeding reduced the SGH prevalence in the treated colonies overtime [8]. An experiment was, therefore, set up to assess the effect of the various treatments on teneral flies screened negative for virus infection so as to eliminate or reduce flies SGH in the progeny.

**Figure 4:**
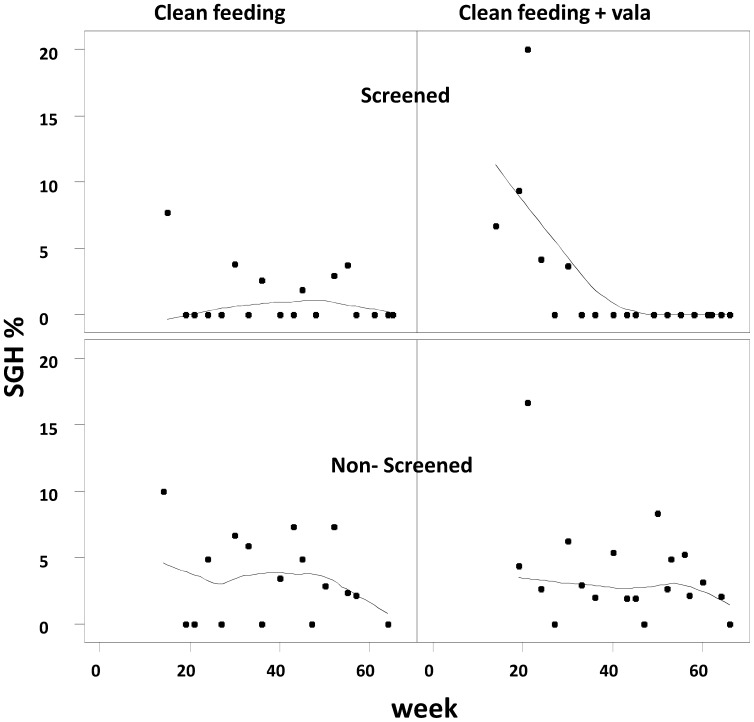
Effect of the combination of clean feeding, screening flies with non-destructive PCR for virus infection and valacyclovir treatment on the expression of SGH syndrome. Screened: teneral flies were tested with non-destructive PCR and only negative flies were used. Non-screened: teneral flies with natural variable virus infection load were used. Flies were fed on blood supplemented or not with 300 µg/ml antiviral drug for 60 days, then virus load was estimated by qPCR.

Combining pre-selection of flies, clean feeding and valacyclovir treatment resulted in the complete elimination of SGH from treated flies in less than 33 weeks.**(** **Fig. 4** **)**, corresponding to approximately 2–3 generations. In the absence of the preselection of negative teneral flies, clean feeding alone or in combination with valacyclovir treatment reduced but did not eliminate the SGH after 60 weeks. The results also showed that although the SGH prevalence in valacyclovir-treated and untreated flies was not significantly different (*P* = 0.712), the difference between screened and unscreened flies was significant (*P*<0.003).

This result are quite important in case the colony manager -due to some difficulties to start a new colony from wild collected flies- needs to establish a new colony with a low virus load from an existing colony infected with the SGHV. Our results indicate that combining pre-selection of flies for SGHV, clean feeding and valacyclovir treatment will significantly reduce the time needed to establish a new *G. pallidipes* colony that is free of symptomatic infections.

### Conclusion

In conclusion, our results show that long term treatment of tsetse colonies with valacyclovir fulfills the four requirements of (i) absence of any significant negative effect on fly survival and productivity, (ii) significant reduction of virus load, (iii) suitable bioavailability when offered to the flies mixed with blood and (iv) affordable price. These requirements have to be considered for use in large scale tsetse rearing facilities.

Important fundamental and practical questions remain to be addressed such as the mode of action of valacyclovir on virus replication, how long the treatment should be maintained to keep the virus load low in the colony and how long the flies can tolerate the drug treatment without hampering on their productivity and mortality. Nevertheless, our data offer a solution to sustain SGHV-infected colonies of *G. pallidipes* and provide the colony manager an alternative to the very long and expensive process of establishing a new colony from field caught flies that probably is not free of virus.

## Materials and Methods

### Strains and Experimental Conditions

All experiments were carried on a *G. pallidipes* colony originating from pupae collected in Tororo, Uganda in 1975 and initially colonized in The Netherlands. These flies were transferred to the Insect Pest Control Laboratory, Seibersdorf, Austria in 1982. The colony adults were fed on heated, defibrinated bovine blood (SVAMAN spol s.r.o., Myjava, 90701, SLOVAKIA) for 10–15 min three times per week using a membrane feeding technique. Each feeding tray with blood and membrane was used to feed up to six sets of cages successively, starting with the youngest flies [24]. The deposited larvae pupate and were incubated at 24°C until emergence. Previous analysis revealed that ca 4–10% of individuals in the colony showed SGH [25].

### Treatments of Flies with Antiviral Drugs and Toxicity Tests (Experiment 1)

Acyclovir (95% purity) and valacyclovir-HCl (96% purity) (both from Molekula, UK), were used to prepare standard solutions in distilled water. Teneral tsetse flies (30 females and 10 males per replicate) were fed either with blood meals containing different concentrations of one compound or blank blood meals in three replicates.

The effect of different concentrations of acyclovir and valacyclovir-HCl (from 30 to1000 µg/ml of blood) on fly fecundity measured as the number of pupae produced per initial female (PPIF), mortality and SGHV copy number as previously described [22] was analyzed by feeding the flies on blood with either drug three times per week for 30 days.

### Long Term Use of Drugs (Experiment 2)

To analyze the impact of the long term use of the drugs on fly biology, SGH prevalence and virus loads, teneral flies (45 females and 15 males in each of three replicates) were fed on blood supplemented with either of the two drugs at 300 µg/ml for 60 days. Pupae from the treated flies were collected and flies emerging from F1 pupae (and subsequent F2 pupae) were treated in a similar way (60 days each generation). Samples of 16 flies/replicate were selected from each treatment and generation for qPCR and the remaining number of flies were dissected and the SGH statue was recorded. To quantify the virus load in experimental flies genomic DNA was extracted from whole flies using DNeasy kit (Qiagen) following the supplier’s instructions and used for qPCR analysis according to previously described conditions [8], [22].

### Clean Feeding and Contaminated Blood

For all tests using the clean feeding system, the feeding trays (membranes and blood) were changed after each set of cages to eliminate the possibility of horizontal virus transmission from one set of cages to another. For the positive control group, three sets of cages of 45 day-old tsetse flies from the standard colony (infected with the virus) were fed with blood first on each membrane to be used in the experiment to deliberately contaminated the blood with virus [8] before each feeding the experimental flies.

### Effect of Antiviral Drugs on the Suppression of SGH Expression and Virus Load (Experiment 3)

To distinguish between the impact of the drugs and the effect of clean feeding on SGH prevalence, teneral flies from the F2 progeny of experiment 2 (with low virus load, see results) were fed either with clean or contaminated blood or with contaminated blood containing valacyclovir at a concentration of 300 µg/ml for 83 weeks. Emerged flies were collected in weekly units, or when not enough flies were collected, the flies emerged over two weeks were collected together. Pupae from the treated and untreated flies were collected on a weekly basis as previously described [26]. The flies were tested for virus load using qPCR and dissected for SGH prevalence when they reached 60 days of age.

### Effect of Combination of Valacyclovir Treatment, Clean Feeding and Screening on the Suppression of SGH Expression (Experiment 4)

A first set of teneral flies screened negative for virus infection using non-destructive PCR [25] were selected and divided into two groups : (i) the first group fed on clean blood and designated as (Screen-Clean feeding), the second group (ii) fed on blood with valacyclovir (300 µg/ml) and designated (Screened-Vala).

A second set of non-screened teneral flies were divided into two groups, (iii) fed on clean blood and designated as (non-screened-clean feeding) and (iv) fed on blood with valacyclovir and designated as (non-screened-Vala). Flies (15 males and 45 females per replicate) were maintained and fed for 60 days and then dissected to record the status of the salivary glands.

### Statistical Analysis

Proportions (mortality and hypertrophy) were angular transformed. Treatments were analyzed by ANOVAR and individual treatment means compared with the Tukey-Kramer HSD test [27]. Analysis was performed using Excel® 13 (Microsoft Corp.), RExcel [28] and R [29].
